# Supplementary material for: Comparative Effects of Flaxseed Sources on the Egg ALA Deposition and Hepatic Gene Expression in Hy-Line Brown Hens
Source: Foods. 2020 Nov 14;9(11):1663. doi: 10.3390/foods9111663 (PMC7696904; doi:10.3390/foods9111663)
Supplement: Supplementary file 1 [file foods-09-01663-s001.pdf]

Table S1. PCA analysis for treatment groups.

		Groups				
		NC	PC	FS	EFM	FSO
**Egg weight**	% Variance	40.64	26.09	16.95	8.89	7.42
	Commulative %	40.64	66.74	83.69	92.58	100.00
**Egg Mass**	% Variance	42.13	20.96	15.17	11.67	10.07
	Commulative %	42.13	63.09	78.26	89.93	100.00
**HDEP**	% Variance	39.79	22.52	17.64	11.55	8.50
	Commulative %	39.79	62.31	79.95	91.49	100.00
**BWG**	% Variance	45.56	22.53	19.63	8.11	4.16
	Commulative %	45.57	68.10	87.73	95.84	100.00
**FI**	% Variance	32.70	21.88	19.09	16.19	10.15
	Commulative %	32.70	54.57	73.67	89.85	100.00
**FCR**	% Variance	39.17	20.07	18.23	12.67	9.86
	Commulative %	39.17	59.25	77.48	90.14	100.00
**Albumin Height**	% Variance	35.84	26.76	16.87	13.71	6.82
	Commulative %	35.84	62.60	79.47	93.18	100.00
**Haugh Unit**	% Variance	33.37	27.26	16.66	15.90	6.81
	Commulative %	33.37	60.63	77.29	93.18	100.00
**TC**	% Variance	59.96	21.53	16.59	1.38	0.54
	Commulative %	59.96	81.49	98.08	99.46	100.00
**TG**	% Variance	57.87	21.24	18.48	1.75	0.64
	Commulative %	57.87	79.12	97.60	99.36	100.00
**LDL-C**	% Variance	50.48	23.60	17.47	6.65	1.80
	Commulative %	50.48	74.08	91.55	98.20	100.00
**HDL-C**	% Variance	47.38	23.73	12.33	11.64	4.90
	Commulative %	47.38	71.12	83.45	95.10	100.00
**VLDL-C**	% Variance	38.82	24.22	17.30	13.50	6.15
	Commulative %	38.82	63.05	80.35	93.85	100.00
**LPL**	% Variance	31.80	24.20	19.74	15.05	9.21
	Commulative %	31.80	56.00	75.74	90.79	100.00
**DHA**	% Variance	68.64	14.06	7.17	6.55	3.57
	Commulative %	68.64	82.70	89.87	96.42	100.00
**Total n-6**	% Variance	80.86	11.03	4.42	2.07	1.61
	Commulative %	80.86	91.89	91.31	98.39	100.00
**Total n-3**	% Variance	91.51	4.83	1.83	1.07	0.76
	Commulative %	91.51	96.34	98.17	99.24	100.00
**n6:n3**	% Variance	90.86	4.18	2.91	1.44	0.614
	Commulative %	90.86	95.05	97.95	99.39	100.00
